# Neurocomputational mechanisms underlying perception and sentience in the neocortex

**DOI:** 10.3389/fncom.2024.1335739

**Published:** 2024-03-05

**Authors:** Andrew S. Johnson, William Winlow

**Affiliations:** ^1^Dipartimento di Biologia, Università degli Studi di Napoli Federico II, Napoli, Italy; ^2^Institute of Ageing and Chronic Diseases, University of Liverpool, Liverpool, United Kingdom

**Keywords:** nerve impulse, physiological action potential, soliton, action potential pulse, computational action potential, reverberatory circuits, perception, sentience

## Abstract

The basis for computation in the brain is the quantum threshold of “soliton,” which accompanies the ion changes of the action potential, and the refractory membrane at convergences. Here, we provide a logical explanation from the action potential to a neuronal model of the coding and computation of the retina. We also explain how the visual cortex operates through quantum-phase processing. In the small-world network, parallel frequencies collide into definable patterns of distinct objects. Elsewhere, we have shown how many sensory cells are meanly sampled from a single neuron and that convergences of neurons are common. We also demonstrate, using the threshold and refractory period of a quantum-phase pulse, that action potentials diffract across a neural network due to the annulment of parallel collisions in the phase ternary computation (PTC). Thus, PTC applied to neuron convergences results in a collective mean sampled frequency and is the only mathematical solution within the constraints of the brain neural networks (BNN). In the retina and other sensory areas, we discuss how this information is initially coded and then understood in terms of network abstracts within the lateral geniculate nucleus (LGN) and visual cortex. First, by defining neural patterning within a neural network, and then in terms of contextual networks, we demonstrate that the output of frequencies from the visual cortex contains information amounting to abstract representations of objects in increasing detail. We show that nerve tracts from the LGN provide time synchronization to the neocortex (defined as the location of the combination of connections of the visual cortex, motor cortex, auditory cortex, etc.). The full image is therefore combined in the neocortex with other sensory modalities so that it receives information about the object from the eye and all the abstracts that make up the object. Spatial patterns in the visual cortex are formed from individual patterns illuminating the retina, and memory is encoded by reverberatory loops of computational action potentials (CAPs). We demonstrate that a similar process of PTC may take place in the cochlea and associated ganglia, as well as ascending information from the spinal cord, and that this function should be considered universal where convergences of neurons occur.

## 1 Introduction

**Figure 1:**
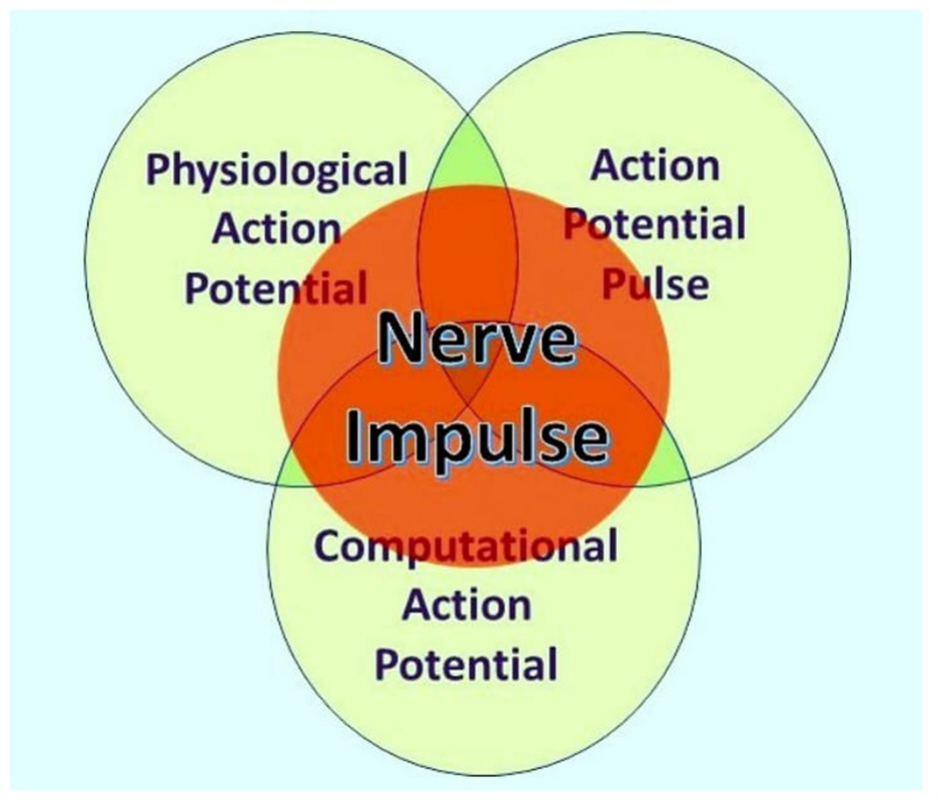
The nerve impulse is an ensemble of three inseparable, concurrent states of the action potential. What an observer will perceive depends on their investigational perspective. The physiological action potential is the orthodox action potential described in detail by Hodgkin and Huxley (1952). The action potential pulse is the mechanical pressure wave for which substantial evidence has been presented elsewhere (Winlow and Johnson, 2021), and the computational action potential is first described by Johnson and Winlow (2017). From Bioelectricity, 3, 161–170, with permission.

According to Ahissar and Assa (2016), “perception of external objects is a closed loop dynamical process encompassing loops that integrate the organism and its environment” and thus provide a probable basis for understanding perception and sentience. Furthermore, neocortical functions are dependent on bidirectional thalamic communications via cortico-thalamic-cortical (CTC) loops interlinked to one another by cortico-cortical (CC) circuits, forming extended chains of loops for communication within the cortex (Shepherd and Yamawaki, 2021). These findings are based on the painstakingly researched physiological connections of neurons within the brains of experimental mammals, such as mice, where many neurons communicate by action potentials and synaptic terminals, either chemical or electrical, although some neurons are spikeless and devoid of action potentials. However, action potentials themselves are known to have three independent functions, namely, communication, modulation, and computation (Winlow and Johnson, 2021), and are the primary elements underlying sentience (Johnson and Winlow, 2018). Furthermore, an individual action potential is an ensemble of three inseparable concurrent states, as shown in Figure 1 (Winlow and Johnson, 2021).

Here, we focus on computational action potentials (CAPs) generated from sensory inputs and how they are computed from sensory areas. We discuss artificial intelligence (AI) and its values as an analogy for brain computation. Computational models of the action potential usually describe it as a binary event, but we have provided evidence that it is a quantum ternary event (Johnson and Winlow, 2021), whose temporal fixed point is the threshold (Johnson and Winlow, 2017, 2019; Winlow and Johnson, 2020), rather than the plastic action potential peak used in other models. In AI, the diffraction of pathways through a network is predetermined by programming each step, but we demonstrate that brain neural network CAPs annul on phase asymmetry, thus leading to distinct network patterning and frequency outputs. We also provide evidence that reverberatory loops of CAPs, each containing 1 trit of information (Johnson and Winlow, 2019, 2021), can provide immediate active memory for every connection in the network, synchronized by phase, thus providing circuit memory. The decoding of the visual cortex is described by first explaining how CAPs affect the patterning of distinct pathways in response to stimulation. A logical examination of the connectome of the visual cortex neurons in response to quantum-phase ternary CAPs demonstrates that timing separates the image from the eye into abstracts, which the neocortex can recognize as objects.

Using the same technique, we show that there is a logical explanation for the actions of the cortex (defined as the convergence of all sensory abstracts). The cortex takes sensory information in the form of abstracts containing all sensory information and therefore contains all the live action, sight, touch, feel, taste, hearing, smell, proprioception, etc., and combines them as action sequences. The cortex may be compared with playing a video with sensation and thus recording live memory. We postulate that the prefrontal neocortex (which we define in terms of connectivity) takes these events and contextualizes them by importance. Thus, the prefrontal neocortex can compare events in the past to the present and can categorize them by context. The brain is, therefore, able to both compare events by time and also by the objects/abstracts within. Further examination of the brain connectome using knowledge of how the brain neural network works should eventually reveal its full functionality.

The cortex is a neural network with random latencies, formed during development from the layered positioning of groups of neurons and synapses with differing connectivity. The neocortex contains many events indexed primarily by time where impactful (repetition and change of context) events become learned. In the frontal lobes of the neocortex, we suggest that present perceptions from other cortical areas are then stored contextually by events and the abstracts within. Therefore, through contextual abstracts, we may view connected past events and imagine future ones. Human thought is a process of comparing the present with past abstractions of what we know of the past and predicting the future from what we know.

## 2 Does the brain work through turing computation?

**Figure 2:**
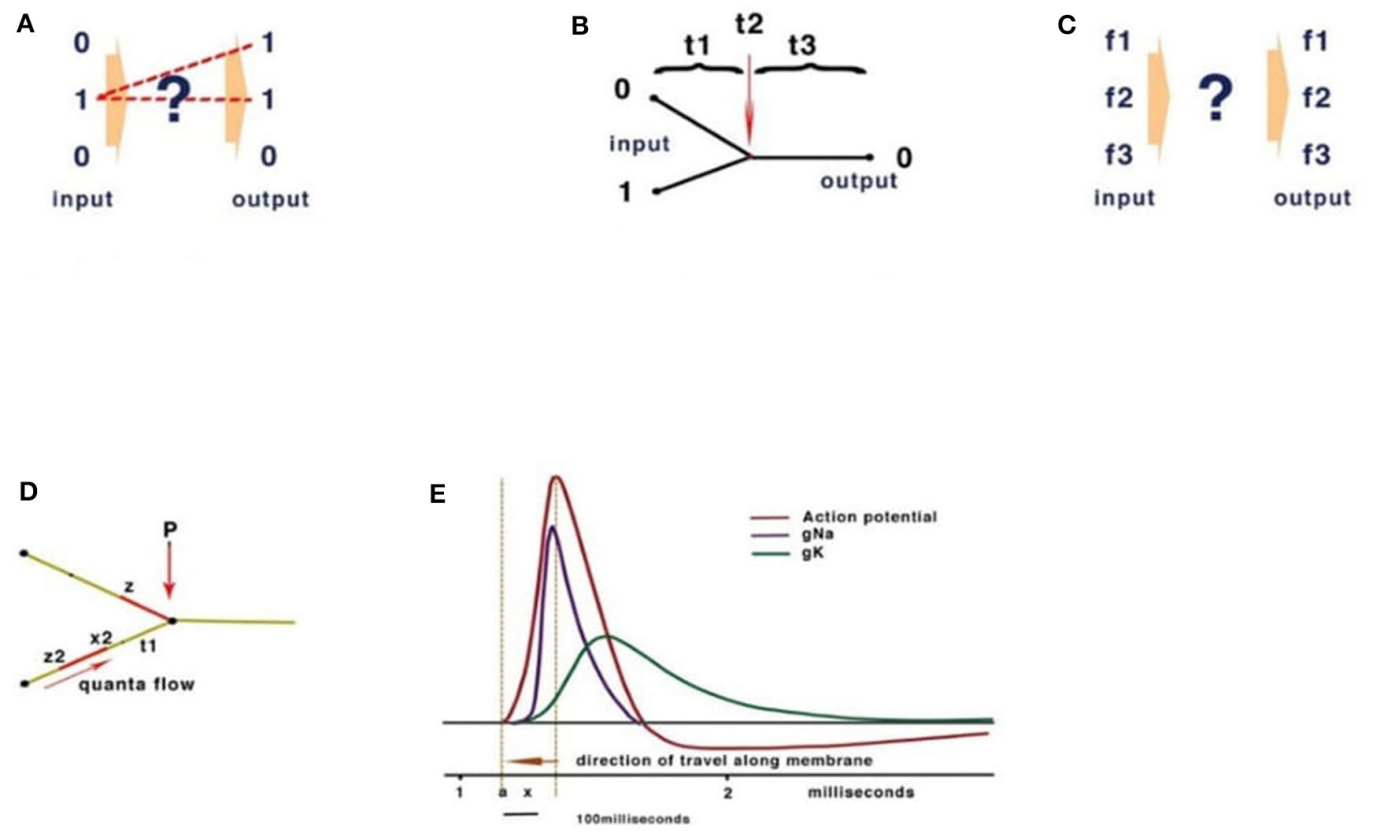
Basic computation rules. **(A)** Inputs must match outputs. **(B)** Turing computers use timing. **(C)** In neural networks, frequency inputs must match their respective outputs. **(D)** The quantum threshold of the action potential (red) propagates to the convergence (P). As the quantum threshold passes the membrane at point P, the membrane becomes refractory, blocking further CAP. The second CAP is therefore destroyed, leading to a reduction of CAP according to phase. **(E)** The action potential showing the threshold area (orange vertical dotted lines) forms the beginning of the quantal soliton with rising and falling currents across the membrane.

Computational theories for the brain rely on analogies with conventional Turing computation. A fundamental feature of Turing computing is the requirement for set-processing (an abstract machine that manipulates symbols on a strip of tape according to a sequential table of rules), usually facilitated by a central timer. However, there is no form of timer in the human brain with the precision to compute action potentials at the rate necessary in the reaction time available (Johnson and Winlow, 2021). Sensory receptors including muscle spindles, rods, cones, etc., all modulate action potential (CAP) frequency in response to the sensation. Similarly, precise activation of muscle is performed by varying frequencies of stimulation; these frequencies are often connected, by convergence, onto interneurons, coding many neuronal outputs into one. However, both the brain and Turing machines comply with basic computation rules. At its simplest, computation is the resolving of unique inputs with appropriate outputs (Figure 2A).

## 3 The computational action potential

Conventional Turing computers process inputs sequentially according to the timing of a gate-directing output and its programming (Figure 2B). The division of time is absolute between operations. The brain, however, computes frequencies rather than time (Figure 2C). To confirm the computational functions of the human brain, it is necessary to fulfill the requirements of computation, explain how this takes place within the limitations of a living neural network, and measure the behavioral properties of neurons and their connections.

Conventional computing, and more recently, artificial intelligence (AI), have evolved because binary chips became commercially available, providing a platform that had not existed previously. The gated mechanism of a transistor is fundamentally different from that of a neuron, with the first acting at nearly light speed and neurons at a leisurely 0.5–10 m/s in unmyelinated axons and up to 120 m/s in myelinated axons. Conventional computing and AI assume that the action potential is binary and that computation is facilitated through the synapses. We disagree and have previously argued that such a mechanism would be too slow, unstable, and error-prone (Johnson and Winlow, 2018) for neurocomputation. It is, therefore, necessary to review the platform of the nervous system from the complexity and positioning of the brain areas down to the fundamental unit of the action potential and to form a hypothesis from the evidence of the demonstrated neuron behavior.

In a conventional computer and in AI, the gating of current is performed by a program defining 0 or 1 (Figure 2B). In Johnson and Winlow's studies (Johnson and Winlow, 2017, 2019), we described how the action potential colliding at convergences can result in similar patterns across a neural network. This form of computation had not been previously described and yet forms an intrinsic property of a neuron. Thus, an action potential can be better described as a quantum pulse with a threshold and peak. As an action potential propagates across a membrane, a refractory period occurs where the pulse has traveled, blocking subsequent CAPs. The mathematics are of quantum processing and depend upon the phase of the colliding CAPs (Figure 2D). In Johnson and Winlow's study (Winlow and Johnson, 2020), we demonstrated that the spike of an action potential is not sufficiently precise to use in computation, but that the threshold had appropriate precision. This supports our view that the CAP is an electromechanical pulse, the APPulse (Johnson, 2015; Johnson and Winlow, 2018, 2021; Winlow and Johnson, 2020), where a pressure pulse soliton (Heimburg and Jackson, 2005; El Hady and Machta, 2015; Perez-Camacho and Ruiz-Suarez, 2017; Ling et al., 2018; Mussel and Schneider, 2019) is the marker for computation rather than the spike. The accuracy, precision, and speed of the CAP are therefore defined by the quantum threshold (Figure 2E). The threshold is the moment of propagation and is at the point where the CAP moves across the membrane. The CAP is therefore composed of a binary signal with a phase element (Johnson and Winlow, 2019, 2021). It is the phase shift when collisions occur (Shrivastava et al., 2018) that changes the frequency of the outputs (Figure 2E). This is very important when considering frequency computation, as the soliton is precise to microseconds. In addition, in Johnson and Winlow's studies (Johnson, 2015; Johnson and Winlow, 2017), we explain how parallel information in a quantum-phase system automatically redacts error, this is especially important when considering noise within the nervous system, and forms concurrent circuit (loop) memory.

## 4 Sensory inputs to the CNS

**Figure 3:**
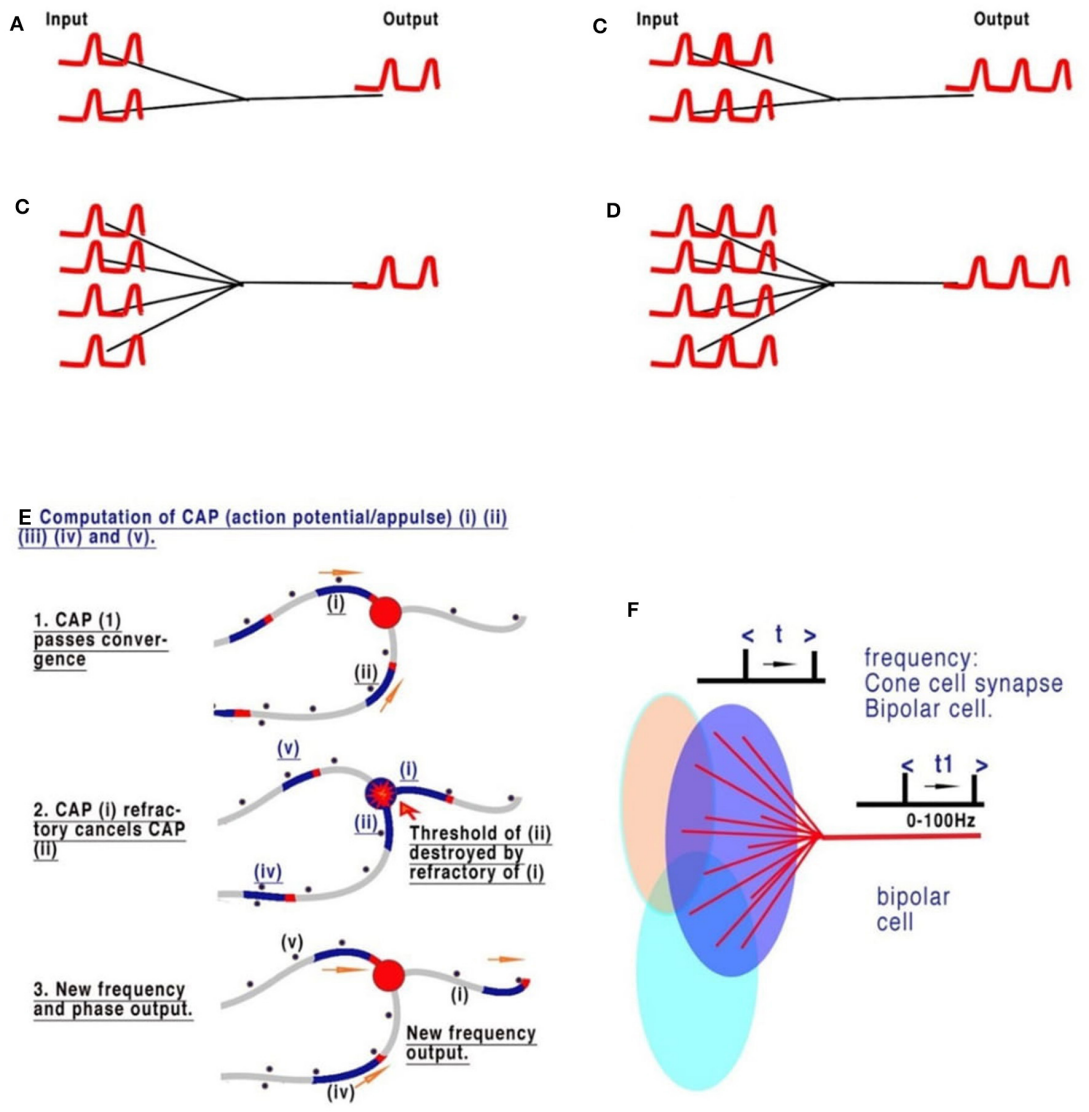
The sensory coding of neurons. **(A–D)** Are deconstructed illustrations of the basic connectivity of a converging neural network. The refractory period of the action potential is dependent on the ability of the membrane to recover. The frequency of action potential is therefore limited in neurons, with most in the range of 0–60 Hz. Observations of the frequencies of input in all converging systems of neurons demonstrate that the frequencies of inputs are proportional to outputs. **(A, B)** Show two and four synapses converging, respectively. Each of the pairs of CAPs arrives in phase and thus neither adds nor subtracts. **(C, D)** Show that an increased frequency is always the result of collective mean sampling. **(E)** Shows details of the CAP cancellation at the convergence. **(F)** Shows the creation of coded sensory gradients in the retinal bipolar cells.

Complex nervous systems have substantial neuronal redundancy and multiple feedback systems to multiple specialized command lines driving central pattern generators (CPGs) (Mussel and Schneider, 2019). The computation of sensory inputs, eventually leading to motor outputs, is most likely achieved by parallel distributed processing, achieved from complex interacting networks in which the CPGs are also embedded. There are innumerable sensory inputs into the mammalian CNS, many of which have been physiologically described but which need to be understood in computational terms, as we have set out in Figure 3, in relation to retinal processing. Figures 3A–D are deconstructed illustrations of the basic connectivity of a converging neural network and the observed results (Johnson and Winlow, 2019). The refractory period of the action potential is dependent on the ability of the membrane to recover. The frequency of action potentials is limited in neurons, with most in the range of 0–100 Hz, but some may generate high-frequency bursts (see below). Observations of frequencies of input in all converging systems of neurons demonstrate that frequencies of inputs are proportional to outputs (Figure 3C), as we commented on in our paper on the neural transactions of the retina (Johnson and Winlow, 2019). The resolution of this mechanism mathematically, within the constraints of a neuron, is the key to understanding its coding. In Johnson and Winlow's study (Johnson and Winlow, 2019), we suggested that the only possible mechanism for this universal effect was interference when the refractory period of the membrane after an initial action potential blocked all others over the same surface for a set period (Figure 3C). After elucidating the mechanism, we then applied this code to the retina. The retina, in common with many other sensory neural networks (auditory, taste, and skin), has similar connectivity of converging activity where regular neural networks from adjacent receptive fields, each supplying a nerve, overlap (Figure 3D). In each case, the initial CAP frequencies are determined by sensory cells (for example, rods and cones). CAPs then converge with interneurons, for example, retinal bipolar cells (RBC). An additive effect of the increasing number of convergences (5 cones to 1 RBC or 20 rods to 1 RBC) has not been observed. Experimentally, the result of the convergence of firing neurons is the mean of the inputs (Figures 3A–D). In Figures 3A–D all the CAPs are in phase, and so the result is a collective mean sampling of the input frequencies. Thus, in the retina, the resulting frequency of the bipolar cell is the mean firing frequency of all its connected light receptors. Therefore, each bipolar cell frequency is the mean representative of a receptive field; where fields overlap, adjacent receptive fields program the light variations and gradients from the bisections (Figures 3A–D). Thus, the bipolar cells effectively code 5–20 photosensitive rods or cones into one bipolar cell. The frequency of CAPs leaving each bipolar cell represents the amount of light activating its respective receptive field. Bipolar cells with adjacent, overlapping fields contain information on light gradients across both fields. Gradient information between three or more overlapping photocells contains all the necessary information by cross-indexing to precisely detect the variations of light across the whole retina. By using gradients, the information from the 5–20 cones is the same as if each cone had its own bipolar cells and optic nerve neurons. Horizontal cells add information from other bipolar cells in adjacent overlapping gradients, coding detail and similarity into the existing frequency. Coding in the ganglion cells follows the same mechanism.

## 5 The visual cortex: what does it do?

**Figure 4:**
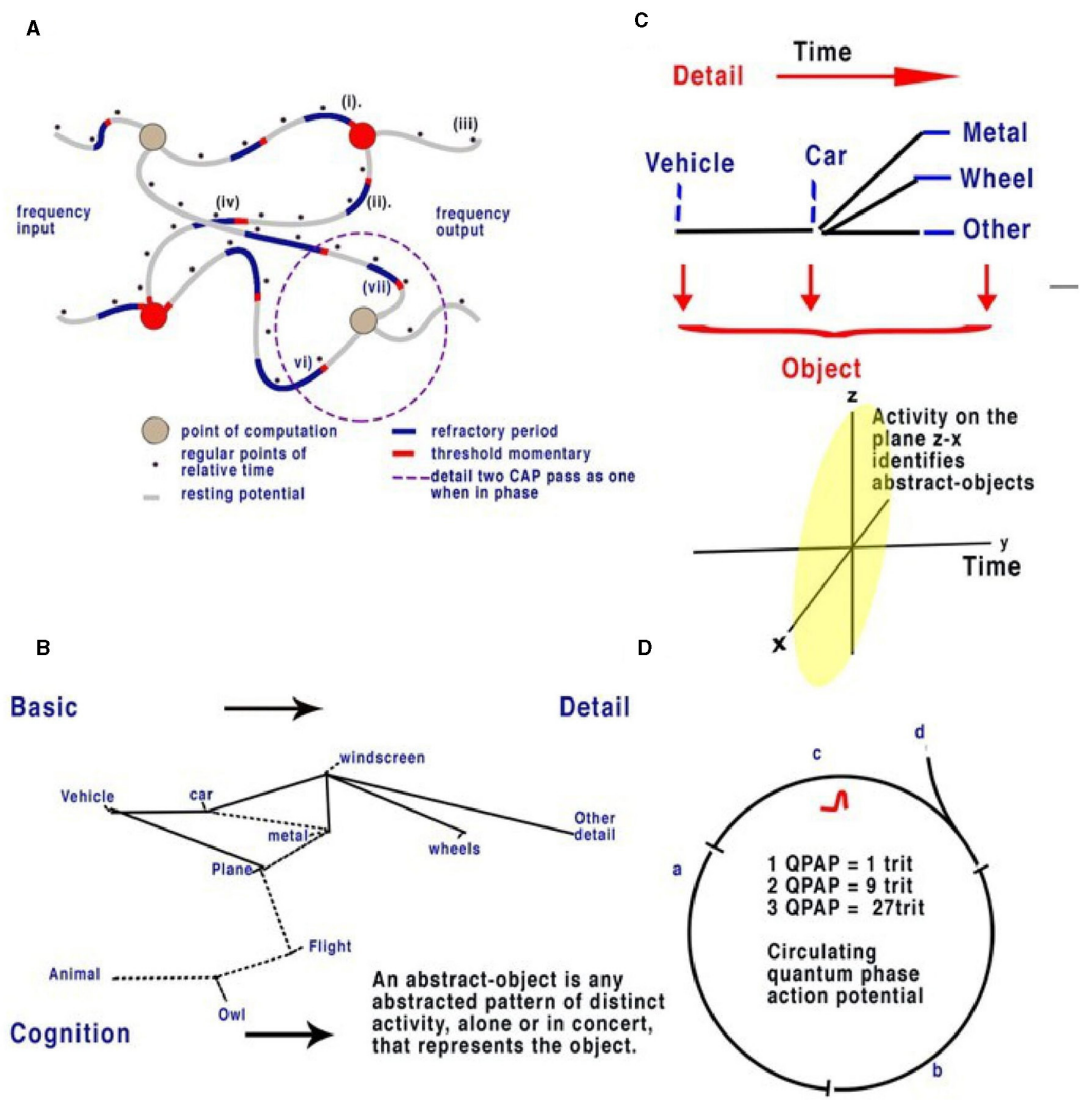
Action potentials travel slowly in comparison to electricity in the unmyelinated neurons, between 0.1 and 1 m/s. In a random neural network, parallel CAPs interfere (Johnson and Winlow, 2019). **(A)** Randomly formed neural network showing quantum APPulse. Frequency inputs match corresponding frequency outputs. **(B)** A contextual network. From the left, a vehicle is formed from a description of some and more details. **(C)** Directionally the object is described by the z-x axis, while the detail is described along the y-axis. **(D)** Three neurons with a circulating CAP contain 1 trit of memory, 2 CAP 9 trits, and 3 CAP 27 trits. A new CAP at “d” will change the phase of the first if it collides with the changing memory. Repetition enables synchronization and error redaction.

**Figure 5:**
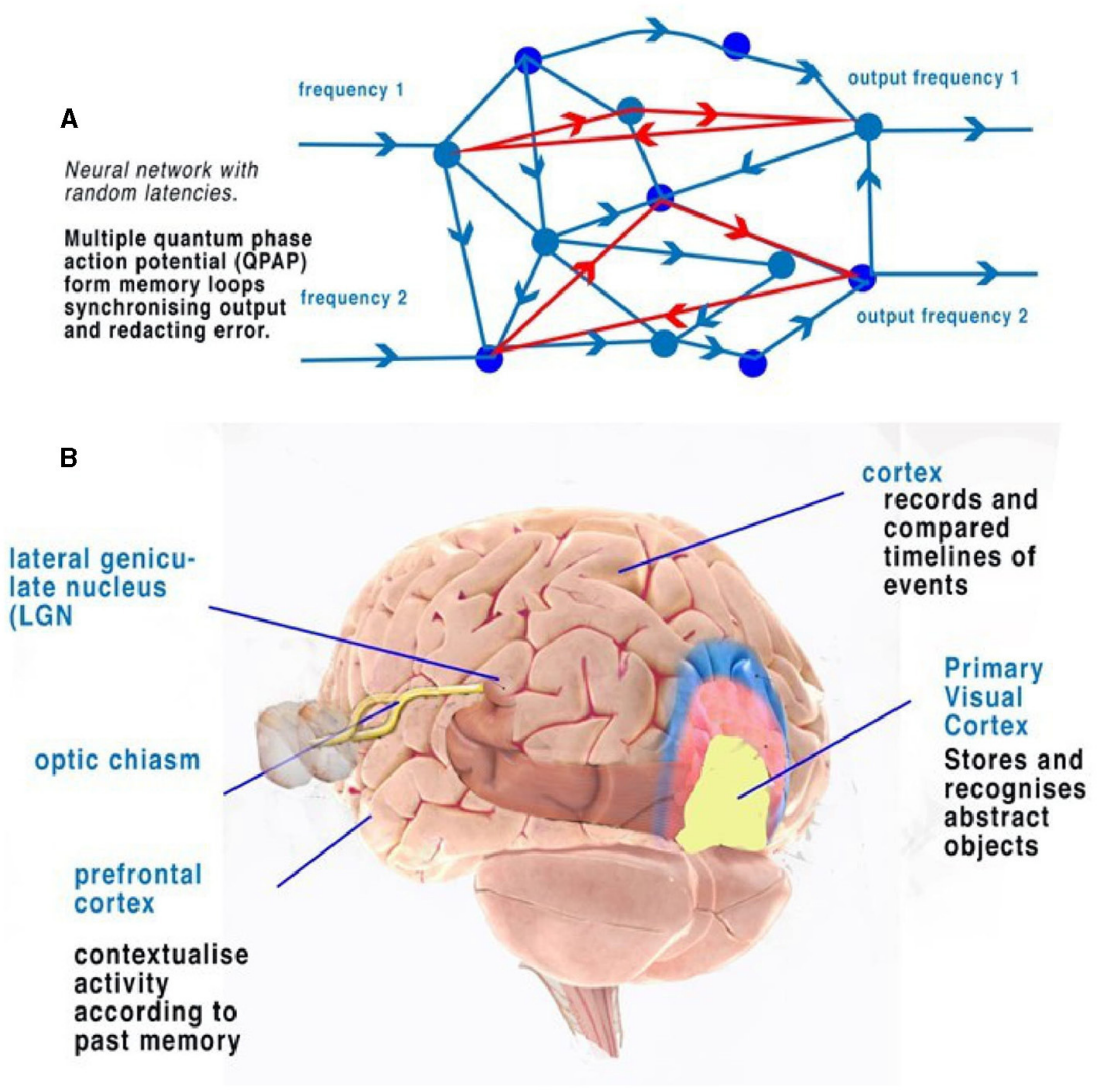
**(A)** An illustration of a random neural network where latencies between nodes are formed from convergence and divergence. Phase ternary computation creates memory circuits or loops of trits within the circulating CAP. **(B)** A simplified illustration of the function of the cortex and neocortex showing areas of progressive perception. Attribution: Society for Neuroscience for the 3D Brain Image.

Coded, parallel, and independent frequencies of CAPs travel along corresponding optic nerve neurons. Before the visual cortex, CAPs are processed by the lateral geniculate nucleus (LGN), which has fast connections to the prefrontal cortex. The LGN can be described within the context of the visual cortex and its function of synchronization. The LGN connects to layer 4 of the primary visual cortex (Espinosa and Stryker, 2012) (Figure 5B). The appearance of the visual cortex is that of neurons of many types grouped into layers with many synapses to many areas, with some neuron types like L5 pyramidal neurons processing many layers. Other types of neurons have been noted with specific morphologies and connectivities that connect within the layers of the visual cortex. Differences in morphology and connectivity are of huge importance in considering computation, as each variation of connection in the connectome will have different latencies. The cortico-thalamic-cortical loops, featuring fast connections and synchronized CAPs, permit the central brain to react to fast-motion objects (like a ball being thrown), without first having to recognize the detailed object. Information from obscured but visible objects as to speed and position are sent simultaneously to the thalamus and to the primary visual cortex. This permits directional recognition of an object and its abstract properties (size, material, and danger) before detailed recognition (texture and weight). Action potentials travel at most 10 m/s in the unmyelinated neurons of the brain. Without the cortico-thalamic-cortical loop, tracking an object, such as a ball, would require geometric calculation. These loops, therefore, provide the brain with not only synchronicity of action potentials but also a method by which activity can be predicted by learned experience. The sensory neural networks of the retina, like the other sensory networks, are regular neural networks permitting the coding of the CAP frequencies, as mentioned. The visual cortex is a layered but otherwise randomly formed network, where both the positioning of synapses and neurons are spaced so that latencies of CAPs are randomly defined. There is also plasticity in the neurons, their connections, and synapses, thus changing CAP latencies over time (Winlow, 1989; Espinosa and Stryker, 2012), which is important in the context of the neural network as it permits memory circuits (Figures 4D, 5A) to be fluid within the network. Plasticity is therefore a functional benefit to the network, permitting potential details of the abstracts to expand.

### 5.1 How does a random neural network in the visual cortex deconstruct/make sense of information from the optic nerves?

In the visual cortex, input frequencies produce defined patterns within the random neural network by interfering with convergences and therefore changing the patterns of output frequencies (Figures 3C, 4A). As the CAPs collide, the patterns they form within the visual cortex will always reflect the output from the retina. The information coded into the optic nerve's 100,000 neurons is a combination of information from adjacent (visual) gradients. The information contains all the details needed to reconstruct the full image from the outline to the detailed similar patterning. In the retina, frequencies from the bipolar cells travel to the retinal ganglion cells (RGC), ensuring that all the information is encoded across all the optic neurons by the collisions and subsequent phase and frequency changes in the RGC. Assuming the optic nerve connections are formed randomly, the output from a few hundred of the optic nerve neurons therefore contains the information of large abstracts – basic color, hue, definition, and change. Each neuron within the optic nerve contains first and foremost the information from its own receptive areas (those directly connected by bipolar cells to cones or rods) (Johnson and Winlow, 2019), forming the main frequency outputs in each case. Information is then combined with the horizontal cells connected to other receptive areas. Frequency is modified by phase by the horizontal cells to reflect the output of all other connected light cells. This computation is phase-dependent and results in a distinct pattern of frequency being computed. This contains all the information regarding similar contexts of light, including hue, color, and saturation, being coded by the location of overlapping similarities. The resultant patterning of frequency codes represents the abstract patterns on the retina. The frequency and change of phase of each optic nerve, therefore, contains, first, the information of its own connected cells and, second, phase changes reflecting patterned similar abstractions (lines, corners, etc.). The information from thousands of inputs, larger abstract shapes, and clearer definitions are thus all coded by frequency and phase change. The selection of two or more optic neurons with overlapping receptive fields contains all the information about objects and abstracts within those fields. As the number of neurons increases, so does the complexity. Thus, the relatively small connection between the LGN and thalamus contains enough information to synchronize large abstracts. As the information passes into the network of the visual cortex, more intricate abstracts are revealed in its pathway. The information from all the neurons gives the complete picture. This mirrors the function of a contextual network (Figure 4B). We propose that the patterning of the visual cortex in response to stimuli is a result of activity being directed by quantum-phase ternary collisions along defined pathways that respond to shapes on the retina. Patterning within the visual cortex has been observed (Kim et al., 2020; Chatterjee et al., 2021), concurring with our hypothesis. The implications of this only become apparent when considering the computation within the spatial dimension and the connectivity of the visual cortex itself.

The computational role of bursting, which can be generated by neurons in the visual cortex (Shai et al., 2015) and in the lateral geniculate nucleus of primates (Martinez-Conde et al., 2002), encoding specific visual stimuli or behavioral states, has been observed elsewhere (Reinagel et al., 1999; Martinez-Conde et al., 2002). Therefore, bursting may be relevant in the computation performed by visual areas. The frequencies of action potentials generated within the LGN (Shai et al., 2015) and primary visual cortex in the L5 pyramidal neurons are likely integrators in the cortical column (Shai et al., 2015). The bursts of frequency timing may therefore be a representation of whole object recognition over time, for example, as abstract shapes from smaller objects form a recognizable image.

Regarding the L5 pyramidal neurons, these are some of the largest neurons spanning many layers where they receive input. Because of the physical dimensions of the membrane and the threshold of action potential quanta being smaller, interference of action potentials may take place over the surface membrane, resulting from the computation of one or more colliding action potentials. Interference patterns of threshold quanta of action potentials collide to modify frequency outputs. In our view, each single pyramidal cell may act as a single computation element, as shown in Figure 2C. In the visual cortex, pyramidal cells connect layers to output. Thus, they are not storing information but computing it. When pyramidal cell activity is decreased during anesthesia (Suzuki and Larkum, 2020), computation and consciousness decrease, while the brain retains memory in the rest of the neural network layers of the cortex.

For an analogy in a contextual network, see Figures 4B, C. In the context of the label “vehicle,” the labels “wheels, windscreen, metal” are positioned so that we can understand without the label what the object is. We can add detail to this to specify a car or a bus, and the initial perception can be added to by specifying detail. If we assume the object is a “vehicle,” then “car, windscreen, etc.,” are all abstracts making up “vehicle.” Each object is an abstract of other objects; each can be combined and subtracted to form other objects. We can also guess what an object is in relation to its abstracts. In addition, if the object is a moving “car” and we have already seen it, we can remember the details from a previous memory. In the visual cortex, a similar deconstruction of recognizable abstracts takes place, each placed within the network. Notice that to see the car moving, it is only necessary to recognize it once; movement can then be tracked separately by the object, and we propose that this occurs in the brain via the LGN.

### 5.2 Spatial memory

In terms of object recognition, the formation of patterns passing through the network can be better understood if the random neural network is drawn according to the timing of CAP. In Figure 4B, the context is gained by cognition as we discover the network reading from left to right so that a complete understanding of the object takes time to examine first the larger object (the vehicle) and then the detail of the vehicle (Figure 4C). Spatially, information being filtered by a network will have directional processing where X and Z coordinates code for each abstract and the Y coordinate for increasing detail. The Y coordinate is important because, in the brain, it is synonymous with the time taken for computation to take place. Y is relative to each individual and their physiology. We propose that the visual cortex functions as a contextual network and can be thought of as a space where information flows directionally, as described in Figure 4. Optic nerve information after entering the visual cortex at the LGN is spread between 100,000 parallel neurons. The small-world random network creates a system where the time taken for CAPs to propagate from a synapse to a convergence or other synapse can be measured in terms of phase differences. Thus, if the threshold precision or latency (proposed to be the beginning of the soliton) is 1 μs (1 × 10^−6^s), then the distance occupied will be 1 × 10^−6^ m. It is the differing latencies of neurons and synapses that create the random neural network. As CAPs interact, any converging CAPs will be annulled, and there will be an effective phase change if CAPs are <1 × 10^−6^s separation. This results in the diffraction of CAPs through the random network, as shown in Figures 4A, B, 5A.

The frequency-modulated parallel output from the optic nerve distributes itself through the LGN-visual cortex, forming a pattern of excitation (Figure 5A). The random architecture ensures that some of the paths are circuits (Figure 5A). In the coding of the retina, patterns formed from the computing frequencies spread according to the timing of the pathways. The optic nerve output passes this information into the visual cortex neural network. The random latencies of the neural connections disperse the output into a distinct pattern of separated, dissimilar abstracts (Figure 4B). CAPs follow discrete network patterns as frequencies pass deeper into the network, reflecting an increasingly complex deconstruction of the pattern of abstracts constituting the image. As information is retrieved, it is passed into the cortex.

If Figure 4C is changed to reflect the direction of time through the random neural network (Figure 4C), we can see that the patterns of activity form abstracts. As patterns progress through the network, the vector Y describes time, and X and Y describe the abstracts and objects, respectively. For a complete image, from the first pattern formation to the last, abstracts of increasing complexity are passed to higher areas, becoming clearer over time. Furthermore, the increase in frequencies may be responsible for bursting (Shai et al., 2015) as synchronization occurs. The implication is that the visual cortex deconstructs the signal into recognizable objects over time that can be sent by parallel CAP frequencies. The LGN passes basic information on the image to the cortex directly, passing on the most rudimentary information on changes. In the visual cortex, the memory of each change is simultaneously formed so that future recognition of abstracts and object labels can occur.

### 5.3 Circuit memory

Circuit memory occurs in a random brain neural network with frequency-modulated inputs and occurs because of recursive CAPs following circuitous routes (Figure 5A). The action potential is a ternary quantum object that, when acting in parallel, can be considered as a trit of information +1, −1, 0 (Johnson and Winlow, 2017, 2018, 2019; Winlow and Johnson, 2021). A single action potential following a circuit passes 1 trit of information; two CAPs store 2 trits, 3^2^; and three trits, 3^3^. This is shown in red in Figure 5A. In a random network, circulatory loops of information can both store memory from changes in phase and redact errors as the network will synchronize and stabilize the output. Theoretically, there is no limit to the number of circulating CAPs or the number of neurons within which the CAPs circulate. In a circulatory path with one CAP, a collision with another entering the circuit will shift the phase of the resultant output, thus changing the memory in the system. In a randomized small-world neural network, these circuits interact and eventually establish a natural synchronized equilibrium with incoming CAPs, thereby maintaining memory by deviating into further patterns. In each case, the quantum ternary CAPs redact errors from the system in parallel; similar abstracts are formed from the inputs, thus colliding and ensuring phase precision through the network (Johnson and Winlow, 2019).

### 5.4 Active circuit memory forms a contextual database

The activity of multiple circuit memory iterations forms a contextual database where every context of the abstract is linked by the circuit memory. In the network, circuit memory is held within specific neuronal pathways that permit CAPs to reverberate, while phase changes record abstracts (Figure 5A). Abstracts have context, which, when combined with others, form recognizable objects. An abstract is therefore a part of the spatial geometry of the neural network. A contextual database is indexed by context with multiple relationships. Firing patterns in the visual cortex therefore represent both the passage of activity through the system and firing from activated circuit memory.

## 6 Discussion

In this study, we have described how quantum-phase CAPs compute and follow distinct patterns in a neural network forming abstracts. This is a fast computational system where chemical synapses have a secondary role in providing latencies, with electrical synapses acting more quickly (Winlow et al., 2017) and in slow inhibition (Johnson and Winlow, 2019). Describing the action as a membrane quantum pulse has the advantage that we can logically explain non-classical neurons that do not produce spikes, as many CNS neurons are spike-less (Roberts and Bush, 1981). Quantum-phase ternary computation is feed-forward, fast, accurate, and error-free. We have also described the coding of the sensory systems in terms of vision provided by the retina, which is a small-world network, and the role of CAPs. In our view, such small-world networks are repeatable and extendable and can be used to predict the detailed functioning of the CNS. Each individual iteration of the random small-world network that forms an abstract representation of an image within the visual cortex can be replicated to produce objects.

The mechanisms described above are universal to neurons, neural networks, and sensory systems in terms of neurocomputation and coding. All of this, indeed, sits in parallel with and accompanies the physiological processes underlying sensory processing. These concepts will, in our view, also underlie the delivery of appropriate motor programs. This implies that phase quantum processing occurs across the membrane of neurons, converging due to the refractory period. The action potential refractory period and its significance were realized by Hodgkin and Huxley (1952) when they described the flow of ions that cause the action potential and realized it was propagated from the threshold. However, the measurements of the speed of processing of information at convergences confirm the beginning of the soliton (Heimburg and Jackson, 2005; El Hady and Machta, 2015; Johnson, 2015; Perez-Camacho and Ruiz-Suarez, 2017; Ling et al., 2018; Chatterjee et al., 2021) as the threshold and the model of the APPulse (Johnson, 2015; Johnson and Winlow, 2018, 2021) as the mechanism of computation.

### 6.1 The visual connectome

Connectivity within the nervous systems is vital; this may include more than just a specific connectome because even individual neurons are capable of computation and computational action potentials are considered quantal. We are entering a new and exciting research phase on nervous function, such as our understanding of the cortico-thalamic-cortical loop in synchronization, as we further investigate the connectome, which appears to be underlain by interacting neurons connected by parallel distributed processing where the classical brain areas of visual, auditory, motor, etc., become understood as spatial connectivity of information.

**Figure 6:**
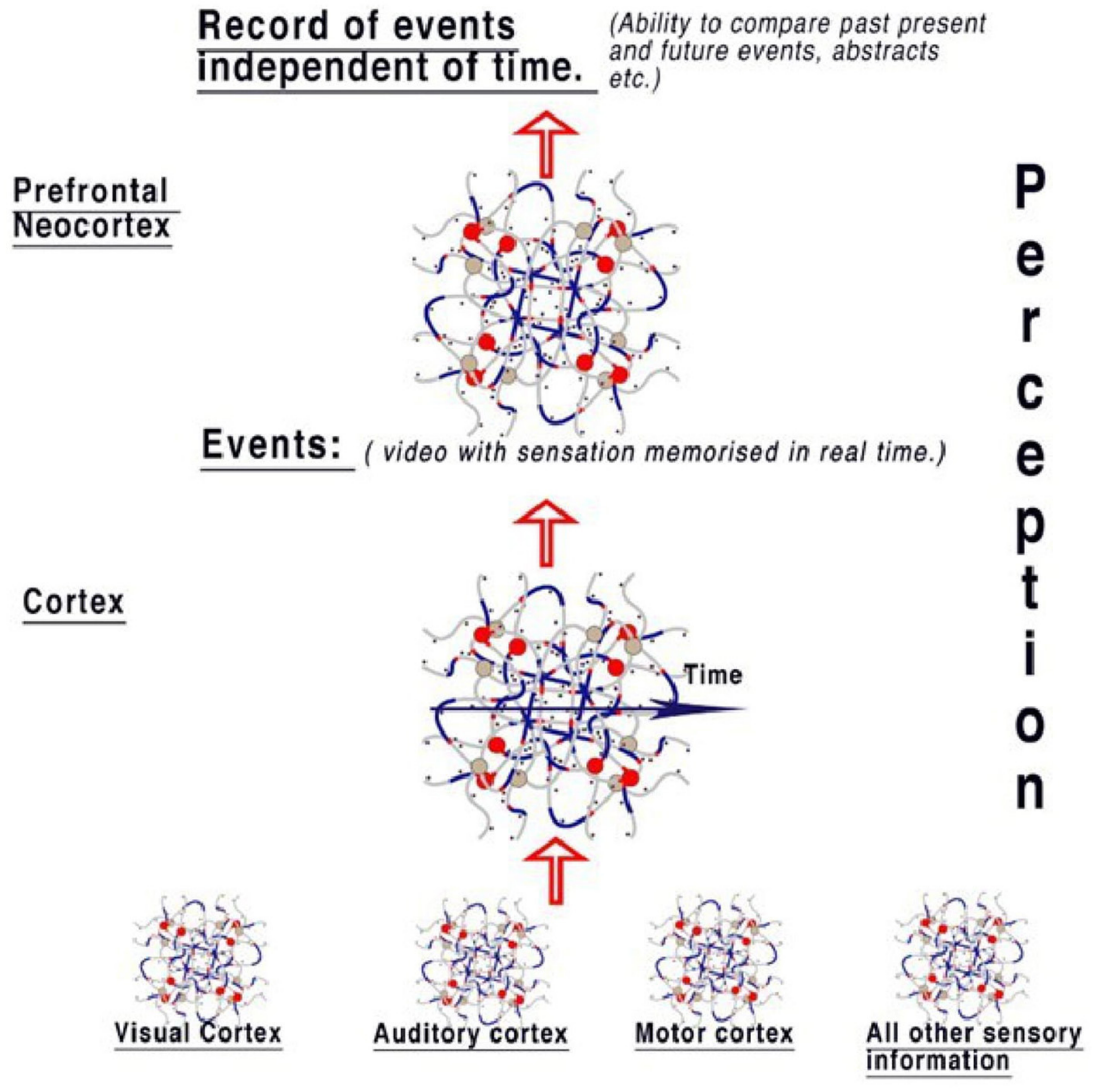
The model of perception from the diffuse brain areas. Sensory areas pass information to the cortex, where events are reconstructed from sensory abstracts and memorized in real-time, therefore creating a “timeline” of events like a video that includes all senses. Information is therefore available to compare events to those already perceived in the cortex. When this information is passed to the neocortex, the event timeline is deconstructed, with the implication that this area is responsible for the ability to think contextually, irrespective of the timeline. This gives the brain the ability to compare objects in relation to the timeline. Therefore, the context between perceptions of events can be examined, for example, by comparing every time an object is seen. An individual perceiving an object can then easily associate it with all events.

We can only speculate about the mechanisms of what happens to the information in spatial abstracts coded in parallel. If the function of the visual cortex is to record and recognize objects and pass on this information, it is logical that the cortex, acting similarly, is storing this information along with all other perceptions from the auditory system, taste, and all other sensory areas to compute the whole perception and memorize it. The cortex is therefore able to process and store present activity as selected events, placing in memory activities of notable changes in the objects and representations forming sensory perception. Events refer to memory from all perceptions—sight, touch, taste, and hearing. This is summarized in Figure 6.

It follows that the prefrontal cortex is computing another level based on these events and both contextualizing and categorizing them, for example, taking the events from the cortex and placing them in a context of importance rather than time. At any time, the neocortex can therefore compare events and objects with all previous similar events and objects, probably in the frontal lobes and objects held within them. The neocortex is therefore able to contextualize current activity according to past memory. In terms of human behavior, this implies that we have a store of memory to refer to for all events in our lives and can compare current situations and memorize them, primarily based on their impact on active circuit memory.

AI networks function according to Turing computing theory and not as brain neural networks. We have shown that the action potential is a quantum ternary structure able to pattern a neural network by frequency modulation and collisions (Kerskens and Pérez, 2022). An AI network has programmed gates that form patterns on each iteration that end in unique output, but that is where the analogy stops. The main difference is that an AI algorithm compares many like abstracts with a query to produce an output of the most likely abstract by probability. There is no evidence that the brain uses probability in selection we show that CAP error and precision correct automatically, and the small-world neural network is of unlimited depth. The decision of which events, objects, and abstracts are activated in the brain for perception is chosen from the context of past to present perception. The small-world random brain neural network can index everything imaginable within a few neurons due to the one trillion synapses and connections in the brain. The system is therefore absolute and determinative, giving a logic of “Yes, no, don't know.” In the network of the visual cortex, components of vision from the retina are split into patterns of abstracts containing information about recognizable objects in descending detail. The conditions under which a recognizable object is not recognized do not exist, as any new object in an image is simultaneously memorized and considered an object. AI networks and the visual cortex both compare abstract representations. However, the “don't know” in AI is determined by probability. By contrast, what we do not know in the visual cortex is produced from detailed synchronous circuit memory. This is important when considering facial recognition, where AI is comparing faces as a probability of recognition. The brain either knows the face or does not. Similarly, with self-driving vehicles, the placement across the road compares similarity and then steers the vehicle on probability. A human subject steering a vehicle is programming motor coordination by comparing the present with all previous driving experiences, road experiences, and any other relevant coordination in their past, in the context of their driving experience.

By storing events contextually, the cortex provides the neocortex with the ability to compare timelines so that events in the present can be compared to the past. The brain has the ability, therefore, to investigate the past and make decisions according to the data presented to us from the context. Decisions in the human brain are therefore made based on an experience from the past, what is happening in the present, and the ability to use past context to predict the future from both.

Currently, we are at an interesting point of advance in neurocomputation. However, studies on neuronal connectivity and deciphering the connectome do not, by themselves, reveal the innermost workings of nervous systems. Furthermore, strong evidence was recently presented to show that there may also be non-classical brain functions due to quantum entanglement between systems. Heartbeat signals were evoked in most parts of the brains of conscious volunteers using nuclear magnetic resonance, as described by Kerskens and Pérez (2022) for the first time. No electrophysiological evidence has ever been found for such connections. In other words, systems of neurons without direct physiological or neurohumoral connections may well be able to influence one another, as we have discussed elsewhere (Winlow and Johnson, 2021; Winlow et al., 2023). Such concepts have been discussed in the past (Johnson, 2007; Larson, 2015), although the idea that quantum mechanics could in part explain higher brain functions was dismissed by some (e.g., Koch, 2006). Obviously, the observations of Kerskens and Pérez (2022) require detailed verification but strongly suggest quantum entanglement between systems whose connections had not been previously observed directly or physiologically identified (Winlow et al., 2023). These findings, if verified, would strongly support our view that the brain uses quantum computation (Johnson and Winlow, 2021), and as a quantum-phase computer, it would be expected of us to generate multiple non-classical connections of this type across the nervous system.

## 7 Conclusion

- In the visual cortex, there are many processes taking place simultaneously that affect the distribution of CAPs to form spatial abstracts: input-coded parallel frequencies, action by synapses, error redaction, and memory circuits.
- We have explained the coding and processing of CAPs into defined visual cortex patterning and given a logical explanation for memory and object recognition. The active memory of reverberatory CAPs within the network is reductive. Every change of pattern is registered in memory, with context supplied by neurons exiting the memory loops.
- Although AI is useful as a tool, we have shown that AI systems are not functionally intelligent, with the danger that their probable answers are accepted as judgments. This is especially true when using insufficient feed data. We have examined AI, which we conclude is an insufficient model of the brain functioning in almost all respects.
- In the retina, we have elucidated the coding, decoding, and function using only the properties of the CAP and the neural connectome. All neurons behave similarly, and we suggest that all areas in the brain function using quantum-phase ternary computation.
- For philosophers, we have answered the question, “Why do physical states give rise to experience?” This also confirms the environmental philosophy of being: we are a sum of our experiences. The confines of the central brain and its connections are created by a few thousand genes. With functioning determined by the positioning and the type of a hundred billion neurons and a trillion synapses, all of which are susceptible to plasticity, genetic variation in a healthy subject is minimized.

## 8 Definitions

QPAP: quantum-phase action potential. Object: any object capable of being recognized by the cortex and may be an abstract of a larger entity. Abstract: any recognizable form that can make up an object. Event: a timeline of objects, sensations, and their interactions. Small-world random neural network: a randomly formed network where latencies between nodes are random, and every node is connected within three degrees of separation.

## Author contributions

WW: Project administration, Supervision, Writing—review & editing. AJ: Conceptualization, Writing—original draft, Writing—review & editing.
